# Fabrication and Conductive Mechanism Analysis of Stretchable Electrodes Based on PDMS-Ag Nanosheet Composite with Low Resistance, Stability, and Durability

**DOI:** 10.3390/nano12152628

**Published:** 2022-07-30

**Authors:** Chengwei Li, Kai Huang, Tingkang Yuan, Tianze Cong, Zeng Fan, Lujun Pan

**Affiliations:** School of Physics, Dalian University of Technology, No. 2 Linggong Road, Ganjingzi District, Dalian 116024, China; chengweili@dlut.edu.cn (C.L.); hk987664186@gmail.com (K.H.); 1424067395@mail.dlut.edu.cn (T.Y.); ctz@mail.dlut.edu.cn (T.C.); fanzeng@dlut.edu.cn (Z.F.)

**Keywords:** stretchable electrodes, PDMS, Ag, contact point, tunneling effect

## Abstract

A flexible and stretchable electrode based on polydimethylsiloxane (PDMS)-Ag nanosheet composite with low resistance and stable properties has been investigated. Under the synergistic effect of the excellent flexibility and stretchability of PDMS and the excellent electrical conductivity of Ag nanosheets, the electrode possesses a resistivity as low as 4.28 Ωm, a low resistance variation in the 0–50% strain range, a stable electrical conductivity over 1000 cycles, and a rapid recovery ability after failure caused by destructive large stretching. Moreover, the conductive mechanism of the flexible electrode during stretching is explained by combining experimental tests, theoretical models of contact point-tunneling effect, and finite element simulation. This research provides a simple and effective solution for the structure design and material selection of flexible electrodes, and an analytical method for the conductive mechanism of stretchable electrodes, which has potential for applications in flexible electronic devices, smart sensing, wearable devices, and other fields.

## 1. Introduction

In recent years—with the rapid development of the flexible electronics [1,2,3], smart wearable devices [4,5,6,7], and smart robotics [8,9]—flexible electronic devices have been receiving more and more attention [10,11,12,13]. As one of the components of these smart electronic devices, stretchable and flexible electrodes are of great importance in structural design, material selection, and electrical conductivity. In terms of structural design, some scientific studies have fabricated stretchable electrodes using materials with poor stretching properties by specific pattern design or structure construction [14,15,16,17], which can be stretched through the deformation of the internal structure of the electrodes, and in this process, the conductive properties of the internal material will not change greatly. Tang et al. developed an Ag wrinkled thin film through the method of pre-stretching and vacuum deposition, and made it into a stretchable electrode. The electrode can maintain a stable electrical conductivity within 100% strain value [18]. Fan et al. proposed a structural strategy that can improve the stretching properties of the material. The research uses the fractal design of the serpentine shapes to topologize the one-dimensional linear structures, which is not stretchable, into loop structures and then into branch-like meshes. As a result, the structure is capable of stretching in multiple directions [19]. Jeong et al. designed a biocompatible metal-patterned porous polydimethylsiloxane (PDMS) electrode with high flexibility and stretchability. In this special structure of the electrode, a large number of pores can disperse the external stress, allowing the electrode to maintain good electrical conductivity during the stretching process [20].

In terms of material selection, some stretchable conductive materials are used to fabricate stretchable electrodes [21,22,23,24]. Wang et al. combined poly (3,4-ethylenedioxythiophene): poly (styrenesulfonate) (PEDOT:PSS) with ionic additives to produce a stretchable electrode. Taking advantage of the good electrical conductivity of PEDOT:PSS and the high fracture strain of the ionic additives, the electrode maintains good electrical conductivity under strain [25]. Zhu et al. fabricated a stretchable fiber-shaped electrode by injecting eutectic gallium–indium (EGaIn) into the hollow elastic yarns. Utilizing the property of EGaIn as liquid alloy at room temperature, the composite yarn has good conductivity even when deformation occurs [26].

In the above two kinds of strategies, conductive materials are required—such as carbon nanotubes, graphene, and other carbon nanomaterials—as well as gold, silver, and other metal materials. As an electrode material, the material is required to own excellent electrical conductivity, not just the ability to conduct electricity. Moreover, the treatment of conductive materials and structures requires some post-processing methods—such as photolithography, ion implantation, thin film deposition, electroplating, UV treatment, etc.—which increases the cost and difficulty of electrode preparation. As a common flexible electrode material, Ag nanomaterials play an important role in many studies, such as 0-dimensional Ag nanoparticles [27,28,29,30], 1-dimensional Ag nanowires [31,32,33,34,35,36], etc. In some studies related to Ag nanomaterial electrodes, it has been found that the contact resistance at junctions between nanomaterials is one of the main issues affecting their electrical conductivity [37]. Aiming at this issue, some post-treatment strategies have been proposed, such as applying heat [38], pressure [39], capillary force [40], chemical reactions [41], laser nano-welding [32,42], nano-joining at the junctions [43], etc.

Herein, in order to fabricate a flexible and stretchable electrode, a composite conductive structure combining Ag nanosheets and PDMS has been investigated. As a metal material with excellent electrical conductivity, Ag is regarded as one of the candidates for electrode material. Compared with Ag nanoparticles and Ag nanowires, Ag nanosheets show the advantages of physical contact stability as electrode materials due to their surface-to-surface contact with each other, which makes them superior to point-to-point and line-to-line contacts. Using PDMS as an elastic carrier for Ag nanosheets can greatly increase the stretchability, allowing them to retain the good conductivity during stretching. In addition, the contact point theory and the tunneling effect theory were applied to reveal the conductive mechanism of the composite electrode during the stretching process. Combined with the method of finite element simulation, the relationship between the structural parameters of the Ag nanosheets inside the electrode and its conductive properties was explored. This study not only designs a flexible and stretchable electrode structure, but also reveals the structure–effect relationship between PDMS-Ag composite and the conductivity, providing a simple and effective solution for the field of flexible electrodes.

## 2. Experimental Section

### 2.1. Materials

The silver glue used in this study was purchased from the Taobao online store of Alibaba Network Technology Co., Ltd. (Hangzhou, China). The silver glue (Model: DJ-F4) has a silver content of 68 wt %, a sheet resistance of less than 0.01 Ω/sq, a density of 1.46–1.48 g/cm^3^ at 25 °C, a silver monomer diameter of less than 10 μm, a viscosity of more than 20,000 CPS, and a weight dilution ratio of 1:0.7–0.8 (conductive agent to diluent). For the preparation of the elastic substrate, a mixture of the base and curing agent for polydimethylsiloxane (PDMS, Dow Corning Sylgard 184) with a mass ratio of 10:1 was prepared.

### 2.2. Fabrication of the Stretchable Electrode

**Figure 1:**
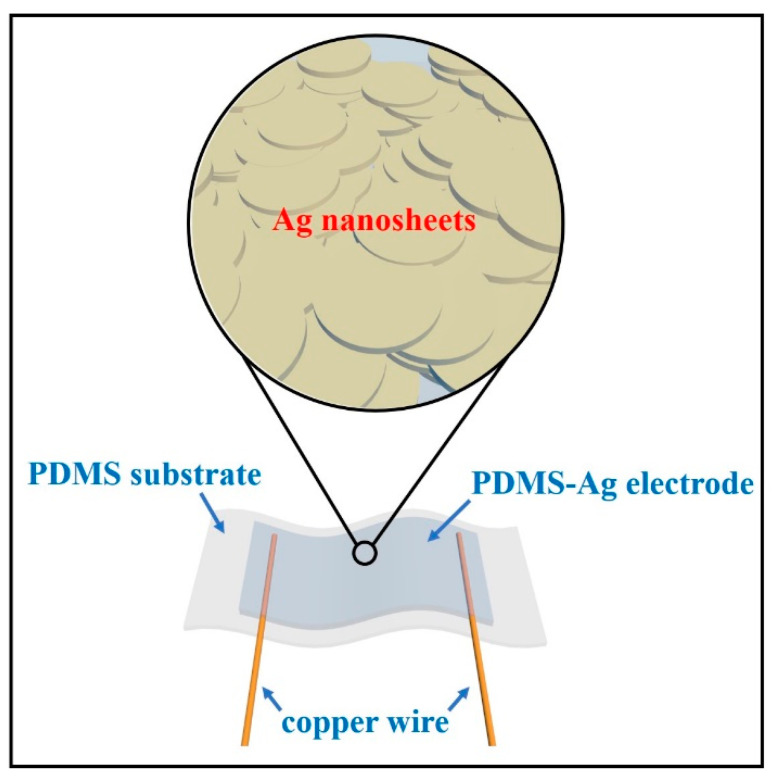
Schematic of the stretchable electrode based on PDMS-Ag nanosheets.

For the fabrication of the stretchable electrode, the PDMS film and a mixture of PDMS and silver glue were used as the flexible substrate and the conductive material, respectively. The detailed preparation process is as follows. The PDMS mixed with the curing agent in advance was dripped into a square mold, and its thickness was controlled to 1 mm, then the air bubbles were removed in a vacuum chamber and the PDMS was dried in an oven at 60 °C for 2 h to obtain a solid PDMS film. Then, the film was cut into a size of 2 cm × 1 cm × 1 mm to obtain a flexible substrate. In order to mix PDMS with Ag nanosheets uniformly, the colloid in silver glue was used as a dispersion medium, thus improving the dispersion of Ag nanosheets in PDMS. In detail, the silver glue and the prepared PDMS were physically mixed in the mass ratios of 1:0, 1:0.3, 1:0.4, 1:0.5, 1:0.6, 1:0.7, and 1:0.8, respectively. The composite PDMS-Ag conductor can be obtained after stirring uniformly. After that, the composite conductor was smeared on the surface of the PDMS substrate, and after removing the air bubbles and drying at 60 °C for 2 h, a composite electrode with a size of about 1 cm × 0.5 cm × 0.2 mm was formed. The schematic of the stretchable electrode is shown in Figure 1.

### 2.3. Characterization and Measurement of the Stretchable Electrode

Optical microscopy (SemiShare SE-4, Shenzhen SemiShare Technology Co., Ltd., Shenzhen, China) and scanning electron microscopy (JCM-5000, JEOL Beijing Technology and Trade Co., Ltd., Beijing, China) were used to observe the micro-structure of the stretchable electrode. A universal material testing machine (YL-S70, Guangzhou Aipeisen Instruments Co., Ltd., Guang Zhou, China) was used to apply a programmed stretching process. A high precision source meter (Agilent B2902A, Agilent Technologies Co., Ltd., Santa Clara, CA, USA) was used to measure the resistance change of the electrode.

### 2.4. Simulation Methods

In order to investigate the electrical conductivity of the composite electrode containing different ratios of Ag and PDMS to help understand the conductive mechanism, the simulation of finite element analysis was performed using the software of COMSOL Multiphysics (version 5.4). Specifically, the potential distribution and current distribution inside the electrodes with different ratios were simulated by constructing a random distribution model of Ag nanosheets.

## 3. Results and Discussion

### 3.1. Morphology Characterization and Basic Electrical Properties of the Stretchable Electrodes

**Figure 2:**
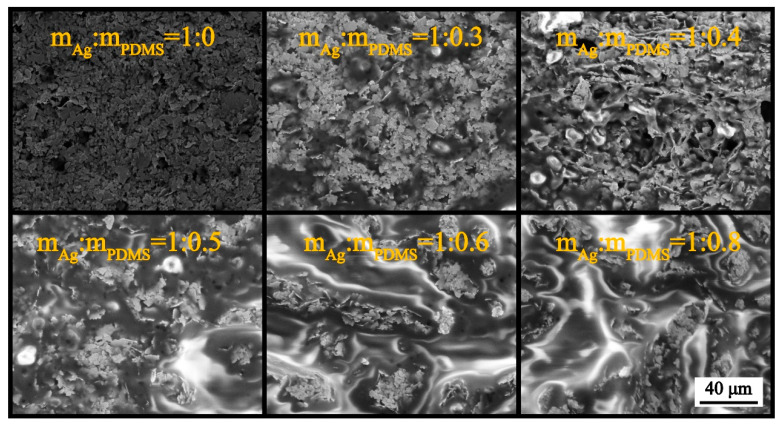
Morphology characterization of the stretchable electrodes based on PDMS-Ag nanosheets with different mass ratios.

In this study, six stretchable electrodes with different mass ratios of silver glue and PDMS were selected as the research objects, under the premise that the mass of silver glue was fixed at 30 mg, and the mass ratios between silver glue and PDMS were set as 1:0, 1:0.3, 1:0.4, 1:0.5, 1:0.6, and 1:0.8, respectively. The morphology characterization of the six stretchable electrodes were observed by SEM (Figure 2) and optical microscopy (Figure S1). It can be seen that the Ag nanosheets are uniformly dispersed in the PDMS. With the gradual increase in the PDMS content, the density of the Ag nanosheets gradually decreases, and the phenomenon that the Ag nanosheets are separated from each other by the gully-like PDMS becomes more and more obvious.

**Figure 3:**
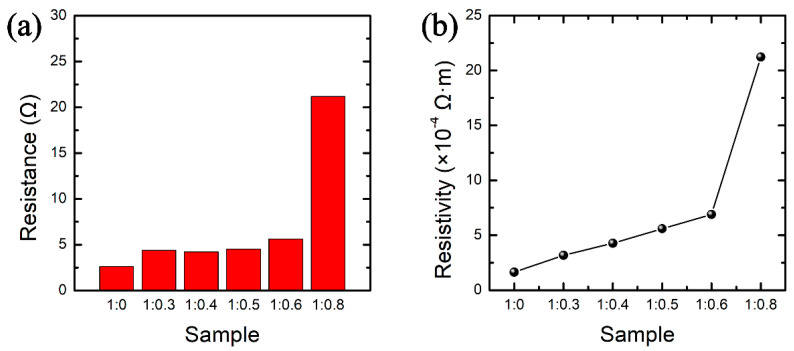
Comparison of (**a**) initial resistance and (**b**) resistivity of PDMS-Ag electrodes with different mass ratios.

According to the statistics of the size of PDMS-Ag composite electrode (Table S1) and the measurement of the initial resistance (Figure 3a), the resistivity of the electrodes with different mass ratios can be calculated, as shown in Figure 3b. It is observed that when the mass ratio of silver glue to PDMS varies from 1:0 to 1:0.6, the initial resistances are all within 6 Ω, and the resistivity increases linearly. When the mass ratio of silver glue to PDMS is 1:0.8, the initial resistance can reach 21.2 Ω, and the resistivity produces a sudden change to 21.3 × 10^−4^ Ω·m. It indicates that with the increase in the PDMS content, PDMS causes a negative effect on the silver conductive pathway, which hinders the conductivity of Ag nanosheets.

### 3.2. Conductive Performance Test of PDMS-Ag Electrodes during Stretching

**Figure 4:**
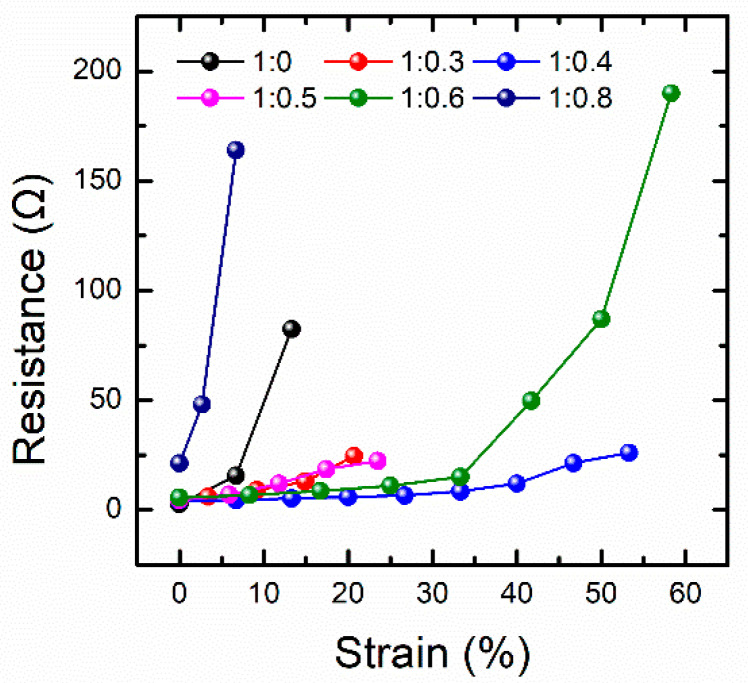
Comparison of the conductive performance of the electrodes with different ratios of silver glue and PDMS during stretching.

The conductive performance of the PDMS-Ag electrodes with different mass ratios during stretching is shown in Figure 4. It is observed that the resistance of all these electrodes increases with the strain variable. By comparing the conductive performances of different electrodes during the stretching process, it can be found that the maximum strain value of the electrodes with a mass ratio of silver glue to PDMS of 1:0 and 1:0.8 is less than 15%, and the resistance shows a rapidly increasing trend. This is because when the mass ratio of silver glue to PDMS is 1:0, the electrode is completely composed of Ag nanosheets, and a large number of Ag nanosheets form an Ag nanosheet film, whose mechanical properties are similar to that of the Ag thin film, and the stretchability is very poor. Therefore, the conductivity of the electrode can be greatly influenced by a small strain. When the mass ratio of silver glue to PDMS is 1:0.8, the PDMS content inside the composite electrode is very high, and the large amount of PDMS separates the limited number of Ag nanosheets from each other, so that the number of initial conductive pathways is very small. When the strain occurs, the original limited number of conductive pathways are further damaged, resulting in the loss of conductivity within 15% of the strain value. Similarly, low contents of PDMS cause the composite electrode to behave Ag film-like property, resulting in the process from integrity to destruction within a relatively small strain range. High contents of PDMS makes for poor conductive ability of the composite electrode, resulting in an easily damaged conductive ability when subjected to a small strain. Importantly, when the mass ratio of silver glue to PDMS is 1:0.4, the conductive performance during stretching is the most stable, and the resistance change is controlled to about 20 Ω within a strain range of more than 50%. Therefore, the composite electrode with this ratio is the optimal electrode under this structural system.

**Figure 5:**
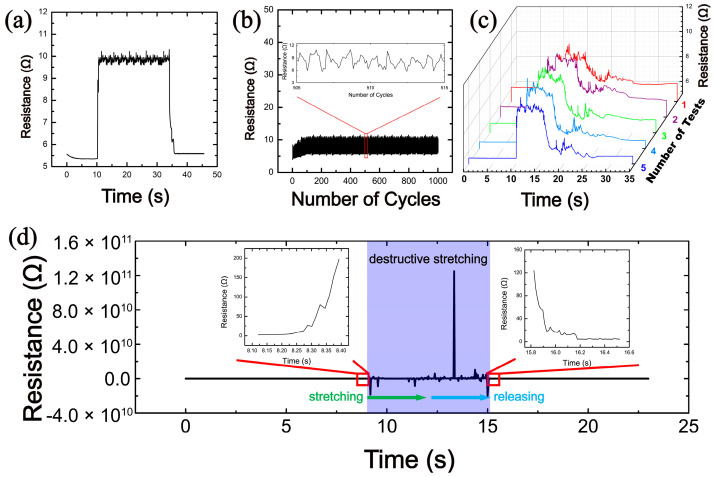
Conductive stability tests of the optimal electrode with a mass ratio of silver glue to PDMS of 1:0.4 during stretching process. Under a strain of 20%, (**a**) sustained stretching test, (**b**) 1000 times cyclic stretching test, and (**c**) intermittent stretching test. (**d**) Destructive stretching-releasing test under a strain of 200%.

For the optimal electrode with a mass ratio of silver glue to PDMS of 1:0.4, the related tests on the stability of the conductivity during the stretching process were performed, as shown in Figure 5. Figure 5a demonstrates the resistance of this electrode at a sustained strain of 20%. It can be seen that the resistance of the electrode increases by only about 4 Ω under a 20% strain, and the resistance is stable and remains about 10 Ω during the process of holding it under the strain of 20% for 25 s. When it stops stretching, the resistance returns to the initial state. It indicates that the electrode ensures stable conductivity under constant static stretching. Figure 5b shows the resistance of the electrode under cyclic stretching. The stress–strain curve in Figure S2 illustrates that the stretching and releasing process of the composite electrode during cyclic stretching is very stable, which provides a guarantee for the resistance measurement during the cyclic test. During 1000 cycles of stretching, the resistance of this electrode consistently varied between 5 and 10 Ω. In addition, the inset shows that the resistance change caused by each stretching is stable. It reflects that the electrode has an excellent fatigue durability to withstand repeated stretching. Figure 5c shows the resistance of the same electrode under intermittent stretching. During the first to fifth stretching tests, the strain was 20% for each stretching process, and the time interval between each test was 4 h. It is illustrated that the resistance change is about 4 Ω during the whole stretching process in each test. In the same stretching test at a fixed interval time, the behavior of the resistance change caused by each stretch is similar. It indicates that the long periods of non-use state do not affect the performance of this electrode, which has excellent stability and reliability in a single stretching test.

It is observed from Figure 5a–c that when a tolerable strain occurs, a small change in resistance appears with the strain. This small resistance change is supposed to originate from the slippage between adjacent Ag nanosheets and a small amount of separation. The slippage and the degree of separation are related to the density and size of the Ag nanosheets. The detailed information will be described later in the finite element analysis.

Figure 5d shows the influence of a destructive large strain on the performance of this electrode. Before the destructive stretching begins, the initial resistance of the electrode is 6 Ω. During the time period of 8–16 s, the electrode undergoes a destructive stretching process with a large strain of 200%, during which the conductive path inside the electrode is completely destroyed, resulting in an open-circuit state of the electrode. After 16 s, the electrode was fully released and the resistance is restored to 6 Ω, ensuring that the electrode could continue to operate normally. The destructive stretching and releasing process under an optical microscopy is shown in Figure S3. The inset of Figure 5d shows the resistance change in a period of time before the start and after the end of the destructive stretching, from which it can be clearly observed that the resistance increases rapidly at the initial state of destructive stretching and decreases rapidly after the electrode is fully released. These results show that this electrode has good robustness and will not permanently fail as a result of a destructive stretch. Furthermore, as a practical flexible sheet, the stability for bending and temperature variation is important. Therefore, the stability tests for the situations of bending operation and temperature variation were performed, as shown in Figure S4. It is observed that the electrode has low resistance both in bending operation and temperature variation, and its resistance changes are small (4 Ω for bending, 2 Ω for temperature variation), confirming the possibility that the electrode can be used under a variety of external stimuli.

### 3.3. Conductive Mechanism Analysis of PDMS-Ag Electrodes

**Figure 6:**
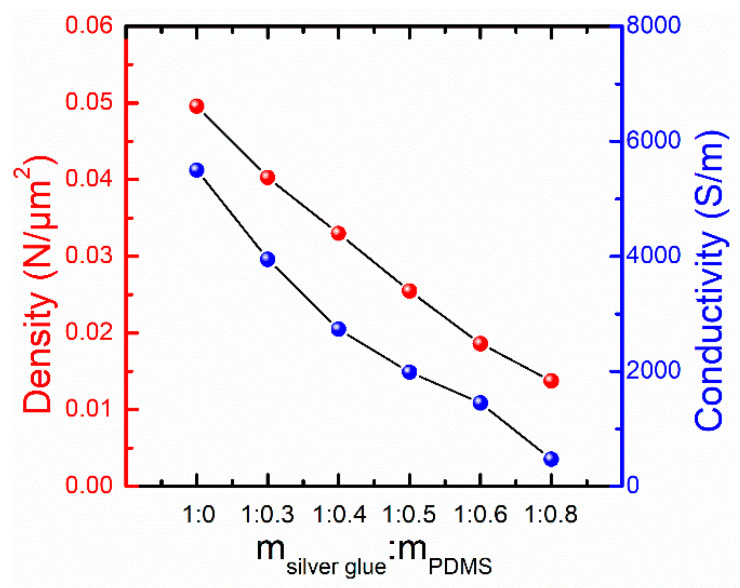
Conductivity of the electrode and density of Ag nanosheets in the electrode containing different ratios of silver glue and PDMS.

In order to explain the conductive mechanism of the PDMS-Ag electrodes, the number of Ag nanosheets was counted according to the SEM images in Figure S5 and the statistical results are listed in Table S2. Based on above results, the density of the Ag nanosheets and the conductivity of the electrodes with different ratios of Ag and PDMS are shown in Figure 6. It is observed that, with the increase in PDMS content, the conductivity of the electrode and the density of Ag nanosheets inside it gradually decrease. This is because the higher the PDMS content is, the more the Ag nanosheets are separated from each other, resulting in a decrease in the number of Ag conductive paths and a decrease in the conductivity. Moreover, the decreasing trends of conductivity and Ag nanosheet density are similar, indicating a positive correlation between them.

According to the above analysis, it is clear that the conductivity of the electrode is directly related to the number of contact points between Ag nanosheets. Therefore, the relationship between the conductivity of electrode and Ag nanosheets can be expressed by the contact point theory as shown in the Supplementary Information [44,45]. After a series of theoretical derivations, the relationship between the resistance and the density of Ag nanosheets can be obtained as

(1) $$\ln\frac{R}{R_{0}}=\alpha^{\prime }\frac{D}{D_{0}}+b^{\prime }$$

where *D* and *D*_0_ are the density and initial density of Ag nanosheets, respectively. Thus, the contact point theory suggests a linear relationship between the relative density (*D*/*D*_0_) of the Ag nanosheets and the logarithm of the relative resistance (*R*/*R*_0_) of the electrode.

**Figure 7:**
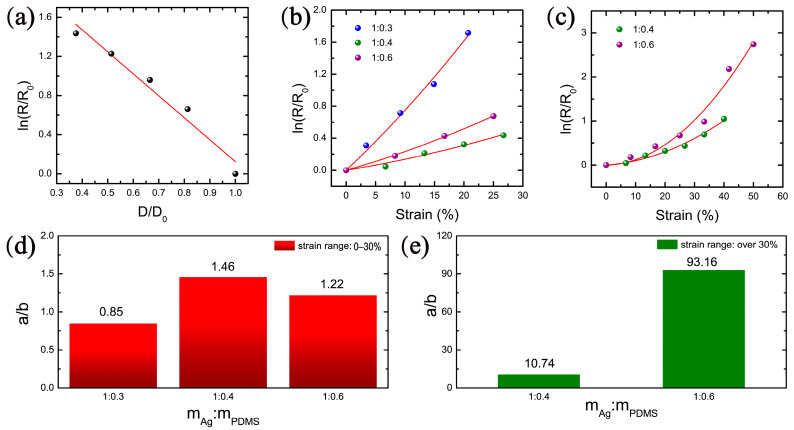
Conductive mechanism analysis of the PDMS-Ag electrodes. (**a**) Contact point theoretical fitting. Tunneling effect theoretical fitting under (**b**) small and (**c**) large strains. Statistics of the proportional coefficients a/b of the tunneling theoretical equation under (**d**) small strain and (**e**) large strain.

The resistance of the electrode is the smallest when the mass ratio of silver glue to PDMS is 1:0, and the resistance increases gradually with the increasing mass ratio of PDMS, which is consistent with the phenomenon that the resistance increases gradually with the increasing strain during the stretching process. Therefore, the density of Ag nanosheets and the resistance of electrode with a mass ratio of silver glue and PDMS of 1:0 are selected as the initial parameters, and the relative density and the relative resistance of electrodes with different mass ratios are calculated. Based on the above analysis, a relationship between relative resistance and relative density is statistically obtained for the electrodes with different mass ratios, as shown in Figure 7a. It can be seen that relative density and relative resistance are consistent with the fitting result (red line) of contact point theory. It indicates that the number of contact points between Ag nanosheets gradually decreases with the increasing of PDMS content, which reflects the conductive state of the electrodes with different mass ratios.

When the stretching process is in progress, the change of electrode resistance not only comes from the change of the contact resistance, but also from the change of the tunneling resistance between the incompletely contacted Ag nanosheets. Therefore, taking the change of tunneling resistance into account, the conductive mechanism of the electrode during stretching can be explained. According to the tunneling theoretical model [46,47,48,49], combined with the actual situation in this study, after a series of theoretical derivations (Supplementary Information), the relationship between tunneling resistance and strain can be obtained as

(2) $$\ln(\frac{R_{t}}{R_{0}})=a\varepsilon^{2}+b\varepsilon$$

Different from the contact point theory, which reflects the state quantity of resistance, the tunneling effect theory reflects the resistance change during the stretching process. In Equation (2), *aε^2^* represents the tunneling resistance term during stretching process, and *bε* represents the contact resistance term of the electrode. Therefore, the total resistance of the electrode can reflect both the contact state inside the electrode and the change process of resistance. The ratio of *a*/*b* can reflect the influence degree of contact resistance and tunneling resistance on the change of electrode resistance, so as to judge the dominant role of them in the contribution of resistance change. When the ratio of *a*/*b* is small, the contact resistance is dominant. Conversely, the tunneling resistance is dominant.

Based on this conclusion, the tunneling effect theory is used to fit the resistance changes of electrodes with a mass ratio of silver glue and PDMS of 1:0.3, 1:0.4, and 1:0.6 during the stretching process within the strain range of 0–30%. The results are shown in Figure 7b. After fitting, the fitting degree (*R*^2^) of the above three electrodes all reach 0.99, indicating that the tunneling theory is in good agreement with the experimental data. According to the fitting results, the proportional coefficients *a*/*b* of the tunneling theoretical equations for these three electrodes are 0.85, 1.46, and 1.22, respectively, as shown in Figure 7d. It indicates that the contact resistance dominates in the electrode with the mass ratio of silver glue to PDMS of 1:0.3. This is because the PDMS content in the electrode is relatively small, and the overall stretchability of the electrode is poor, a small strain can undergo the process from complete to destruction of the internal conductive network of the electrode. Therefore, the resistance change of this electrode is mainly manifested by the contact resistance. The proportional coefficients *a*/*b* of the electrodes with the mass ratio of silver glue to PDMS of 1:0.4 and 1:0.6 are larger than that of 1:0.3, indicating that the tunneling effect on the resistance gradually increases with the decrease in the density of Ag nanosheets.

Since the maximum strain value of electrode with a mass ratio of silver glue and PDMS of 1:0.3 is only 20%, the tunneling theory is fitted for the electrodes with a mass ratio of 1:0.4 and 1:0.6 in the wider range of 0–50%, and the fitting results and the statistics of the proportional coefficients a/b of the tunneling theoretical equation are shown in Figure 7c,e respectively. It is observed that in the strain range of 0–50%, the resistance changes of both electrodes during stretching are consistent with the tunneling effect theory, and the proportional coefficients a/b of the tunneling theoretical equation of the two electrodes are 10.74 and 93.16, respectively. In the stretching process from small to large, the proportional coefficients a/b of the electrode with a mass ratio of 1:0.6 increases from 1.22 to 93.16, which is much larger than that of the electrode with a mass ratio of 1:0.4 from 1.46 to 10.74. It indicates that, with the further increase in the strain, the influence of tunneling resistance becomes more obvious, which is caused by the separation of a large number of Ag nanosheets from each other under large strains. For the proportional coefficients a/b of the tunneling theoretical equation, the electrode with a mass ratio of 1:0.6 is significantly more than that with a mass ratio of 1:0.4. This is because the electrode with a mass ratio of 1:0.6 has a high PDMS content, resulting in a low number of initial conductive pathways. The separation between Ag nanosheets caused by stretching is obvious, leading to a high tunneling resistance. However, the electrode with a mass ratio of 1:0.4 keeps a large number of Ag nanosheets in contact with each other during the stretching process, thus maintaining a relatively stable conductivity under a large strain. Therefore, the electrode with a mass ratio of 1:0.4 is the optimal ratio for the PDMS-Ag electrode.

### 3.4. Finite Element Analysis of the PDMS-Ag Electrodes

**Figure 8:**
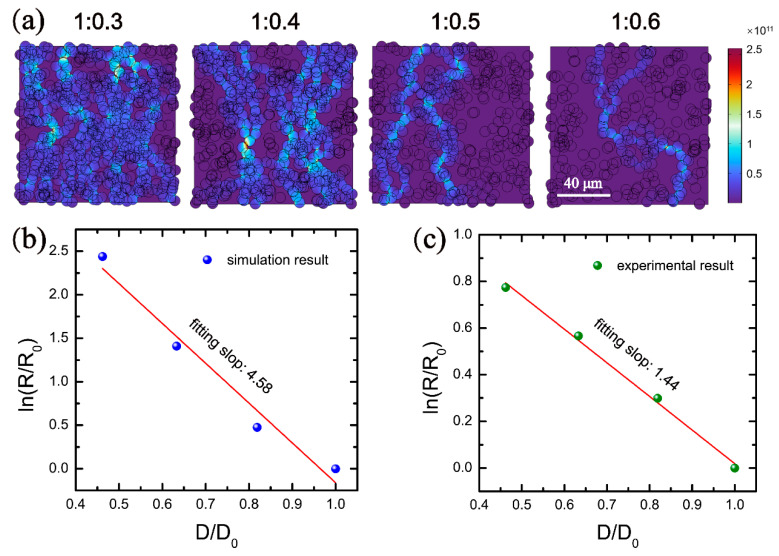
Comparison of simulation and experimental results. (**a**) The current density distribution inside the composite electrode under the mass ratios of Ag nanosheets to PDMS of 1:0.3, 1:0.4, 1:0.5, and 1:0.6, respectively. Contact point theoretical fitting of (**b**) simulation and (**c**) experimental results.

In order to obtain a deeper and comprehensive understanding of the conductive mechanism and the influence factors, the PDMS-Ag electrode is modeled by the method of finite element analysis. Due to the fact that the thickness of the electrodes is much smaller than the size of the surface region, it can be considered that the Ag nanosheets are less distributed in the direction perpendicular to the plane and mainly concentrated in the surface region. Moreover, in the direction perpendicular to the surface, the Ag nanosheets in contact with each other can be regarded as a whole. Therefore, for the purpose of simplifying the model, the three-dimensional conductive model can be replaced by the two-dimensional in-plane conductive model, which plays a dominant role. Firstly, the size of the Ag nanosheets in the SEM image of the electrode is counted, as shown in Figure S6. According to the statistical results, the average radius of the Ag nanosheets is 3.912 μm. Therefore, the radius of the Ag nanosheet is set to 4 μm during modeling. Then, the physical model of the PDMS-Ag composite film is established by combining the statistics of the density and size of the Ag nanosheets in the electrode, and an electric potential of 0.1 V is applied to the model, the potential distribution is shown in Figure S7. Based on this model, the current density distribution inside the composite electrode was simulated under the mass ratios of Ag nanosheets to PDMS of 1:0.3, 1:0.4, 1:0.5, and 1:0.6, respectively, as shown in Figure 8a. It is clear that the current density decreases gradually with the increase in the PDMS content, this trend is consistent with the experimental phenomenon. The simulation data under different relative densities of Ag nanosheets are fitted using the contact point theory, and the fitting results are found to be consistent with the theory, as shown in Figure 8b. Comparing the simulation result with the experimental data (Figure 8c), it is found that the relative resistance change of the simulation result is large than that of the experimental result, and the fitting slope of the simulation result is 4.58, which is larger than that of the experimental data of 1.44. The reason for the difference between the simulation and experimental results may come from the fact that some measured Ag nanosheets are covered by other adjacent Ag nanosheets during the counting process, resulting in a smaller measured value of the Ag nanosheets than the actual value.

**Figure 9:**
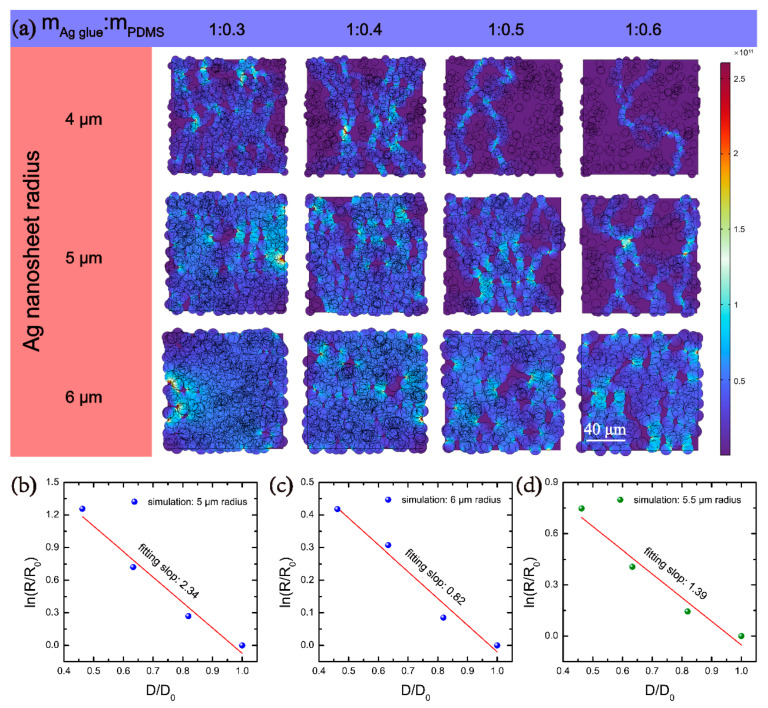
Simulation of the conductivity of electrodes with different Ag nanosheet radii. (**a**) Comparison of the current density distribution when the radius of Ag nanosheets is 4, 5, and 6 μm, respectively. Contact point theoretical fitting for the Ag nanosheet radius of (**b**) 5 μm, (**c**) 6 μm, and (**d**) 5.5 μm, respectively.

Considering the deviation in the measurement process of Ag nanosheet size, the conductivity of the electrode with different Ag nanosheet sizes was simulated to modify the model based on the contact point theory. Therefore, the radius of the Ag nanosheets was increased from the initial 4 μm to 5 μm and 6 μm, respectively, and the current density distribution was simulated, as shown in Figure 9a. It can be seen from the simulation results that with the increase in the Ag nanosheet size, the current density and conductive pathways in the electrodes with the same mass ratio increase gradually, and correspondingly, the resistance gradually decreases. Contact point theory was used to fit the Ag nanosheets with a radius of 5 μm and 6 μm, and the fitting results are shown in Figure 9b,c. According to the fitting results of these two situations, the fitting slopes are 2.34 and 0.82, the logarithm of the relative resistances are 1.26 and 0.42, respectively. This is because with the increase in the Ag nanosheet size, the contact area between the mutually stacked Ag nanosheets becomes larger. During the stretching process, Ag nanosheets are more difficult to separate from each other, resulting in a decrease in the change rate of the number of conductive pathways and a smaller resistance change in the electrode. Therefore, a large Ag nanosheet size is beneficial to improve the conductive stability of the electrode during stretching. From the above discussion and the experimental results in Figure 8c, it can be seen that the fitting slope of the contact point theory of the experimental data is 1.44, and the change of logarithm of relative resistance is 0.77. Comparing the experimental results in Figure 8c with the simulation results in Figure 9b,c, it can be seen that both the fitting slope and the change of logarithm of relative resistance of the experimental results are between the fitting results for the Ag nanosheet radius of 5 μm and 6 μm. Following this trend, an Ag nanosheet radius between 5 and 6 μm would exist. Under this radius, the slope and the change of logarithm of relative resistance fitted by the contact point theory are similar to the experimental results in Figure 8c. By simulating and fitting different Ag nanosheet radii between 5 and 6 μm, it is found that when the Ag nanosheet radius is 5.5 μm, the fitting slope and the change of logarithm of relative resistance are 1.39 and 0.75, respectively, which are very close to the experimental results. Therefore, the average radius of the Ag nanosheets used in the experiment can be inferred to be around 5.5 μm through this method. The simulation results modify the measured values of the Ag nanosheet size based on SEM images.

Therefore, the flexible and stretchable electrodes can be fabricated by mixing PDMS with Ag nanosheets, and the conductive stability of the composite electrodes during stretching can be regulated by adjusting the density and size of the Ag nanosheets.

## 4. Conclusions

In summary, a PDMS-Ag-based composite electrode was prepared. Taking advantage of the properties of good flexibility and stretchability of PDMS and good conductivity of Ag, the electrode has the ability to maintain good conductivity during stretching. By comparing the conductivity of different electrodes, it is found that the electrode with a mass ratio of PDMS and silver glue of 1:0.4 has the best stability during stretching. The electrode has an initial resistance of only about 5 Ω, a maximum strain value of over 50% in normal operation, and a limited resistance change within 20 Ω during the whole stretching process. In addition, it can maintain stable conductivity during constant stretching process; withstand more than 1000 cycles of stretching tests; and under the state of the electrode failure caused by destructive large stretching, the conductivity can be rapidly recovered after the stress is released. The conductive mechanism of the electrode is explained by combining the contact point theory and the tunneling theory. The change rule of the conductivity under different Ag nanosheet densities and the conductivity of the electrodes under different mass ratios during stretching process are investigated. The dominant role of contact resistance and tunneling resistance is judged by the value of the proportional coefficients a/b of the tunneling theoretical equations. Moreover, the simulation method is used to explain the effect of Ag nanosheet size on the conductivity, and the average radius of the Ag nanosheet inside the electrode is inferred to be 5.5 μm by simulation. This study provides a simple and effective method to fabricate a flexible and stretchable electrode, and has potential in flexible electronics, wearable devices, and other fields.

## Supplementary Materials

The following supporting information can be downloaded at: https://www.mdpi.com/article/10.3390/nano12152628/s1, Figure S1: optical microscopy observation of the stretchable electrodes with different mass ratios silver glue and PDMS; Table S1: The statistics of the size of PDMS-Ag composite electrodes; Figure S2: The stress-strain curves of 100 continuous cyclic stretching-releasing test; Figure S3: The surface morphology of the composite electrode during the process of destructive stretching and releasing under an optical microscopy; Figure S4: The stability of the electrode under different external stimuli, (a) bending test, (b) temperature variation test; Figure S5: The statistics on the number of Ag nanosheets by counting method; Table S2: The statistics on the density of Ag nanosheets; Figure S6: The statistical size of the Ag nanosheet. (a) SEM image of the statistical area. (b) The statistical results of the Ag nanosheet radii; Figure S7: Modeling process of the electrode through the finite element simulation. The schematic of (a) model building and (b) potential distribution.

## References

[1] Wang C.Y., Xia K.L., Wang H.M., Liang X.P., Yin Z., Zhang Y.Y. (2019). Advanced Carbon for Flexible and Wearable Electronics. Adv. Mater..

[2] Zhao J., Li N., Yu H., Wei Z., Liao M.Z., Chen P., Wang S.P., Shi D.X., Sun Q.J., Zhang G.Y. (2017). Highly Sensitive MoS2 Humidity Sensors Array for Noncontact Sensation. Adv. Mater..

[3] Li C.W., Zhang Y.F., Yang S.T., Zhao H.T., Guo Y., Cong T.Z., Huang H., Fan Z., Liang H.W., Pan L.J. (2022). A flexible tissue-carbon nanocoil-carbon nanotube-based humidity sensor with high performance and durability. Nanoscale.

[4] Li C.W., Yang S.T., Guo Y., Huang H., Chen H., Zuo X.Q., Fan Z., Liang H.W., Pan L.J. (2021). Flexible, multi-functional sensor based on all-carbon sensing medium with low coupling for ultrahigh-performance strain, temperature and humidity sensing. Chem. Eng. J..

[5] Liao M., Ye L., Zhang Y., Chen T.Q., Peng H.S. (2019). The Recent Advance in Fiber-Shaped Energy Storage Devices. Adv. Electron. Mater..

[6] Lv T., Yao Y., Li N., Chen T. (2016). Wearable fiber-shaped energy conversion and storage devices based on aligned carbon nanotubes. Nano. Today.

[7] Tadesse M.G., Mengistie D.A., Chen Y., Wang L.C., Loghin C., Nierstrasz V. (2019). Electrically conductive highly elastic polyamide/lycra fabric treated with PEDOT:PSS and polyurethane. J. Mater. Sci..

[8] Wang Z.P., Wang Y.S., Chen Y.J., Yousaf M., Wu H.S., Cao A.Y., Han R.P.S. (2019). Reticulate Dual-Nanowire Aerogel for Multifunctional Applications: A High-Performance Strain Sensor and a High Areal Capacity Rechargeable Anode. Adv. Funct. Mater..

[9] Zhang C., Liu S.Y., Huang X., Guo W., Li Y.Y., Wu H. (2019). A stretchable dual-mode sensor array for multifunctional robotic electronic skin. Nano. Energy.

[10] Yu L.T., Yeo J.C., Soon R.H., Yeo T., Lee H.H., Lim C.T. (2018). Highly Stretchable, Weavable, and Washable Piezoresistive Microfiber Sensors. Acs. Appl. Mater. Inter..

[11] Lv Z.S., Luo Y.F., Tang Y.X., Wei J.Q., Zhu Z.Q., Zhou X.R., Li W.L., Zeng Y., Zhang W., Zhang Y.Y. (2018). Editable Supercapacitors with Customizable Stretchability Based on Mechanically Strengthened Ultralong MnO2 Nanowire Composite. Adv. Mater..

[12] Trung T.Q., Kim C., Lee H.B., Cho S.M., Lee N.E. (2020). Toward a Stretchable Organic Light-Emitting Diode on 3D Microstructured Elastomeric Substrate and Transparent Hybrid Anode. Adv. Mater. Technol..

[13] An B.W., Gwak E.J., Kim K., Kim Y.C., Jang J., Kim J.Y., Park J.U. (2016). Stretchable, Transparent Electrodes as Wearable Heaters Using Nanotrough Networks of Metallic Glasses with Superior Mechanical Properties and Thermal Stability. Nano. Lett..

[14] Hwang H., Kim D.G., Jang N.S., Kong J.H., Kim J.M. (2016). Simple method for high-performance stretchable composite conductors with entrapped air bubbles. Nanoscale Res. Lett..

[15] Hong H.C., Chen C.M. (2014). Design, Fabrication and Failure Analysis of Stretchable Electrical Routings. Sens. Basel.

[16] Qaiser N., Damdam A.N., Khan S.M., Bunaiyan S., Hussain M.M. (2021). Design Criteria for Horseshoe and Spiral-Based Interconnects for Highly Stretchable Electronic Devices. Adv. Funct. Mater..

[17] Webb R.C., Bonifas A.P., Behnaz A., Zhang Y.H., Yu K.J., Cheng H.Y., Shi M.X., Bian Z.G., Liu Z.J., Kim Y.S. (2013). Ultrathin conformal devices for precise and continuous thermal characterization of human skin. Nat. Mater..

[18] Tang J., Guo H., Zhao M.M., Yang J.T., Tsoukalas D., Zhang B.Z., Liu J., Xue C.Y., Zhang W.D. (2015). Highly Stretchable Electrodes on Wrinkled Polydimethylsiloxane Substrates. Sci. Rep..

[19] Fan J.A., Yeo W.H., Su Y.W., Hattori Y., Lee W., Jung S.Y., Zhang Y.H., Liu Z.J., Cheng H.Y., Falgout L. (2014). Fractal design concepts for stretchable electronics. Nat. Commun..

[20] Jeong G.S., Baek D.H., Jung H.C., Song J.H., Moon J.H., Hong S.W., Kim I.Y., Lee S.H. (2012). Solderable and electroplatable flexible electronic circuit on a porous stretchable elastomer. Nat. Commun..

[21] Chen W., Li Y.D., Li R.Q., Thean A.V.Y., Guo Y.X. (2018). Bendable and Stretchable Microfluidic Liquid Metal-Based Filter. IEEE Microw. Wirel. Co..

[22] Cao Y., Morrissey T.G., Acome E., Allec S.I., Wong B.M., Keplinger C., Wang C. (2017). A Transparent, Self-Healing, Highly Stretchable Ionic Conductor. Adv. Mater..

[23] Park M., Park J., Jeong U. (2014). Design of conductive composite elastomers for stretchable electronics. Nano. Today.

[24] Ladd C., So J.H., Muth J., Dickey M.D. (2013). 3D Printing of Free Standing Liquid Metal Microstructures. Adv. Mater..

[25] Wang Y., Zhu C.X., Pfattner R., Yan H.P., Jin L.H., Chen S.C., Molina-Lopez F., Lissel F., Liu J., Rabiah N.I. (2017). A highly stretchable, transparent, and conductive polymer. Sci. Adv..

[26] Zhu S., So J.H., Mays R., Desai S., Barnes W.R., Pourdeyhimi B., Dickey M.D. (2013). Ultrastretchable Fibers with Metallic Conductivity Using a Liquid Metal Alloy Core. Adv. Funct. Mater..

[27] Hong G.B., Luo Y.H., Chuang K.J., Cheng H.Y., Chang K.C., Ma C.M. (2022). Facile Synthesis of Silver Nanoparticles and Preparation of Conductive Ink. Nanomaterials-Basel.

[28] Kim K.K., Choi J., Kim J.H., Nam S., Ko S.H. (2022). Evolvable Skin Electronics by In Situ and In Operando Adaptation. Adv. Funct. Mater..

[29] Nguyen H.L., Jo Y.K., Cha M., Cha Y.J., Yoon D.K., Sanandiya N.D., Prajatelistia E., Oh D.X., Hwang D.S. (2016). Mussel-Inspired Anisotropic Nanocellulose and Silver Nanoparticle Composite with Improved Mechanical Properties, Electrical Conductivity and Antibacterial Activity. Polymers-Basel.

[30] Yeo J., Kim G., Hong S., Kim M.S., Kim D., Lee J., Lee H.B., Kwon J., Suh Y.D., Kang H.W. (2014). Flexible supercapacitor fabrication by room temperature rapid laser processing of roll-to-roll printed metal nanoparticle ink for wearable electronics application. J. Power Sources..

[31] Wu X.L., Wang S.Y., Luo Z.W., Lu J.X., Lin K.W., Xie H., Wang Y.H., Li J.Z. (2021). Inkjet Printing of Flexible Transparent Conductive Films with Silver Nanowires Ink. Nanomaterials-Basel.

[32] Lee P., Lee J., Lee H., Yeo J., Hong S., Nam K.H., Lee D., Lee S.S., Ko S.H. (2012). Highly Stretchable and Highly Conductive Metal Electrode by Very Long Metal Nanowire Percolation Network. Adv. Mater..

[33] Vo T.T., Lee H.J., Kim S.Y., Suk J.W. (2020). Synergistic Effect of Graphene/Silver Nanowire Hybrid Fillers on Highly Stretchable Strain Sensors Based on Spandex Composites. Nanomaterials-Basel.

[34] Won P., Park J.J., Lee T., Ha I., Han S., Choi M., Lee J., Hong S., Cho K.J., Ko S.H. (2019). Stretchable and Transparent Kirigami Conductor of Nanowire Percolation Network for Electronic Skin Applications. Nano. Lett..

[35] Jung J., Cho H., Yuksel R., Kim D., Lee H., Kwon J., Lee P., Yeo J., Hong S., Unalan H.E. (2019). Stretchable/flexible silver nanowire electrodes for energy device applications. Nanoscale.

[36] Cho J.H., Kang D.J., Jang N.S., Kim K.H., Won P., Ko S.H., Kim J.M. (2017). Metal Nanowire-Coated Metal Woven Mesh for High-Performance Stretchable Transparent Electrodes. Acs. Appl. Mater. Inter..

[37] Ge Y.J., Duan X.D., Zhang M., Mei L., Hu J.W., Hu W., Duan X.F. (2018). Direct Room Temperature Welding and Chemical Protection of Silver Nanowire Thin Films for High Performance Transparent Conductors. J. Am. Chem. Soc..

[38] Song T.B., Chen Y., Chung C.H., Yang Y., Bob B., Duan H.S., Li G., Tu K.N., Huang Y., Yang Y. (2014). Nanoscale Joule Heating and Electromigration Enhanced Ripening of Silver Nanowire Contacts. Acs. Nano..

[39] Tokuno T., Nogi M., Karakawa M., Jiu J.T., Nge T.T., Aso Y., Suganuma K. (2011). Fabrication of silver nanowire transparent electrodes at room temperature. Nano. Res..

[40] Celano T.A., Hill D.J., Zhang X., Pinion C.W., Christesen J.D., Flynn C.J., McBride J.R., Cahoon J.F. (2016). Capillarity-Driven Welding of Semiconductor Nanowires for Crystalline and Electrically Ohmic Junctions. Nano. Lett..

[41] Lu H.F., Zhang D., Cheng J.Q., Liu J., Mao J., Choy W.C.H. (2015). Locally Welded Silver Nano-Network Transparent Electrodes with High Operational Stability by a Simple Alcohol-Based Chemical Approach. Adv. Funct. Mater..

[42] Lee J., Lee P., Lee H., Lee D., Lee S.S., Ko S.H. (2012). Very long Ag nanowire synthesis and its application in a highly transparent, conductive and flexible metal electrode touch panel. Nanoscale.

[43] Lee J., Lee P., Lee H.B., Hong S., Lee I., Yeo J., Lee S.S., Kim T.S., Lee D., Ko S.H. (2013). Room-Temperature Nanosoldering of a Very Long Metal Nanowire Network by Conducting-Polymer-Assisted Joining for a Flexible Touch-Panel Application. Adv. Funct. Mater..

[44] Li C.W., Pan L.J., Deng C.H., Wang P., Huang Y.Y., Nasir H. (2017). A flexible, ultra-sensitive strain sensor based on carbon nanocoil network fabricated by an electrophoretic method. Nanoscale.

[45] Li C.W., Pan L.J., Deng C.H., Cong T.Z., Yin P.H., Wu Z.L. (2017). A highly sensitive and wide-range pressure sensor based on a carbon nanocoil network fabricated by an electrophoretic method. J. Mater. Chem. C.

[46] Xu S., Rezvanian O., Peters K., Zikry M.A. (2013). The viability and limitations of percolation theory in modeling the electrical behavior of carbon nanotube-polymer composites. Nanotechnology.

[47] Dang Z.M., Jiang M.J., Xie D., Yao S.H., Zhang L.Q., Bai J.B. (2008). Supersensitive linear piezoresistive property in carbon nanotubes/silicone rubber nanocomposites. J. Appl. Phys..

[48] Chen L., Chen G.H., Lu L. (2007). Piezoresistive behavior study on finger-sensing silicone rubber/graphite nanosheet nanocomposites. Adv. Funct. Mater..

[49] Li C.W., Pan L.J., Deng C.H., Wang P. (2016). CNC-Al2O3-Ti: A new unit for micro scale strain sensing. Rsc. Adv..

