# Supplementary material for: Fabrication and Conductive Mechanism Analysis of Stretchable Electrodes Based on PDMS-Ag Nanosheet Composite with Low Resistance, Stability, and Durability
Source: Nanomaterials (Basel). 2022 Jul 30;12(15):2628. doi: 10.3390/nano12152628 (PMC9370586; doi:10.3390/nano12152628)
Supplement: Supplementary file 1 [file nanomaterials-12-02628-s001.zip › nanomaterials-1823303-supplementary.pdf]

nanomaterials

Supplementary Information

Fabrication and Conductive Mechanism Analysis of Stretchable Electrodes Based on PDMS-Ag Nanosheet Composite with Low Resistance, Stability, and Durability

Chengwei Li, Kai Huang, Tingkang Yuan, Tianze Cong, Zeng Fan, and Lujun Pan *

School of Physics, Dalian University of Technology, No. 2 Linggong Road, Ganjingzi District, Dalian 116024, China; chengweili@dlut.edu.cn (C.L.); 987664186@qq.com (K.H.); 1424067395@qq.com (T.Y.); ctz@mail.dlut.edu.cn (T.C.); fanzeng@dlut.edu.cn (Z.F.)
* Correspondence: lpan@dlut.edu.cn

1. The optical microscopy observation of the six stretchable electrodes

Figure S1 shows the optical microscopy observation of the stretchable electrodes with different mass ratios of silver glue and polydimethylsiloxane (PDMS), *i.e.*, 1:0, 1:0.3, 1:0.4, 1:0.5, 1:0.6 and 1:0.8, respectively. It can be seen that with the increase of the PDMS content, the Ag conductive pathways (black part) gradually decrease and the PDMS insulating islands (white part) gradually increased.

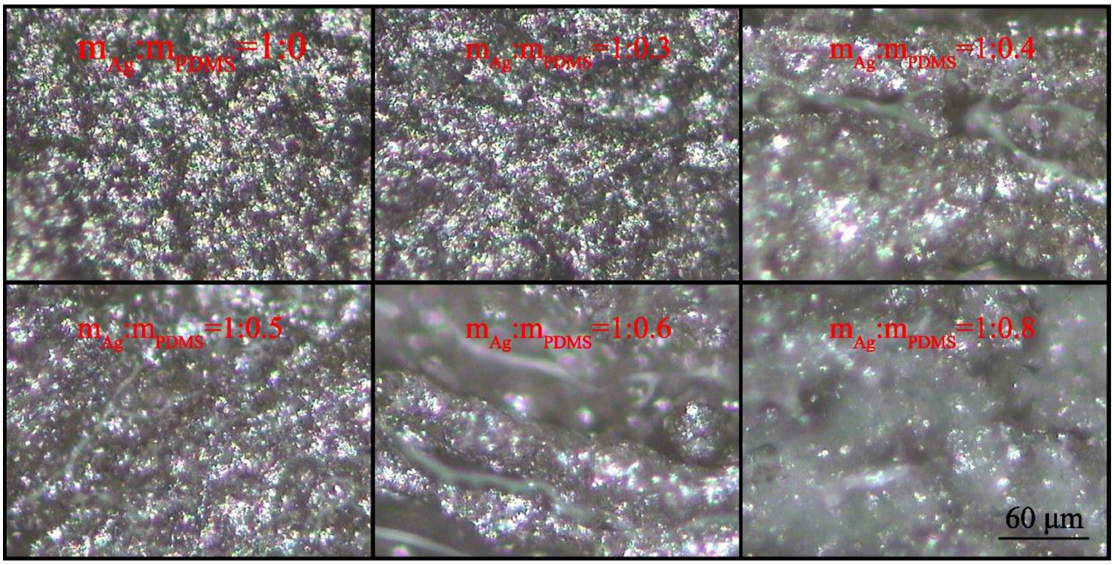

Figure S1. optical microscopy observation of the stretchable electrodes with different mass ratios silver glue and PDMS.

2. The statistics of the size of PDMS-Ag composite electrodes

Table S1. The statistics of the size of PDMS-Ag composite electrodes.

$m_{silver\ glue}$:m_{PDMS}	Length (mm)	Width (mm)	Thickness (mm)
1:0	15.0	5.5	0.172
1:0.3	11.0	3.5	0.227
1:0.4	10.0	4.0	0.255
1:0.5	10.0	4.0	0.311
1:0.6	10.0	3.8	0.324
1:0.8	13.0	6.0	0.217

3. The stress-strain curves of cyclic stretching-releasing test

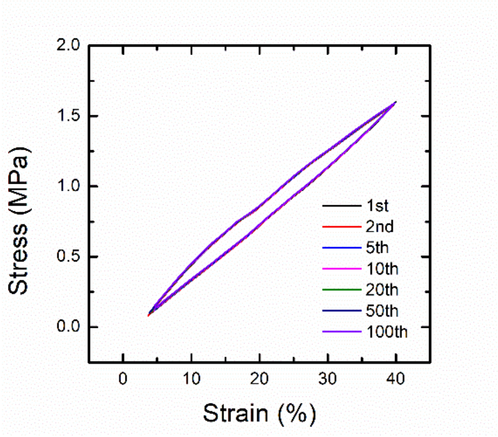

Figure S2. The stress-strain curves of 100 continuous cyclic stretching-releasing test.

4. The destructive stretching and releasing process under an optical microscopy

Figure S3 shows the destructive stretching and releasing process under an optical microscopy. It is observed that the insulation area appears on the surface of the electrode after a destructive stretching, and the surface returns to the initial state after stress release.

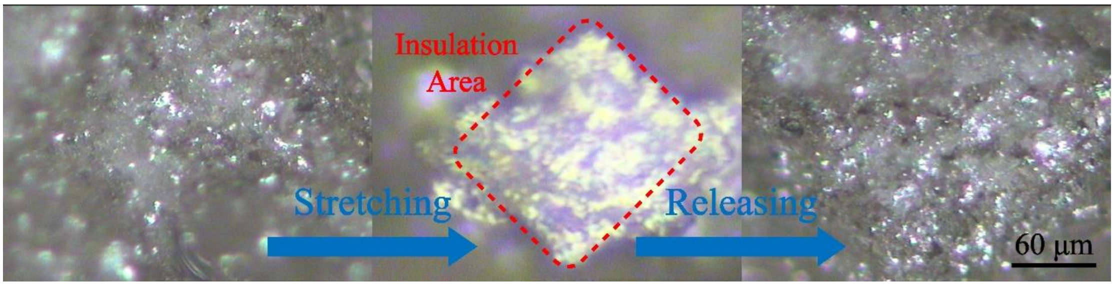

Figure S3. The surface morphology of the composite electrode during the process of destructive stretching and releasing under an optical microscopy.

5. The stability tests of bending operation and temperature variation

The bending test and temperature variation test have been performed, as shown in Figure S4. In the bending test (Figure S4a), the distance between the two ends of the electrode decreases by 50% during bending, and the resistance of the electrode changes by only 4 Ω during the whole process, reflecting the good bending stability of the electrode. In the temperature variation test (Figure S4b), the temperature changes between 0 and 80 °C, and the resistance change of the electrode is kept within 2 Ω during the whole process, which indicates that the electrode has good variable temperature stability.

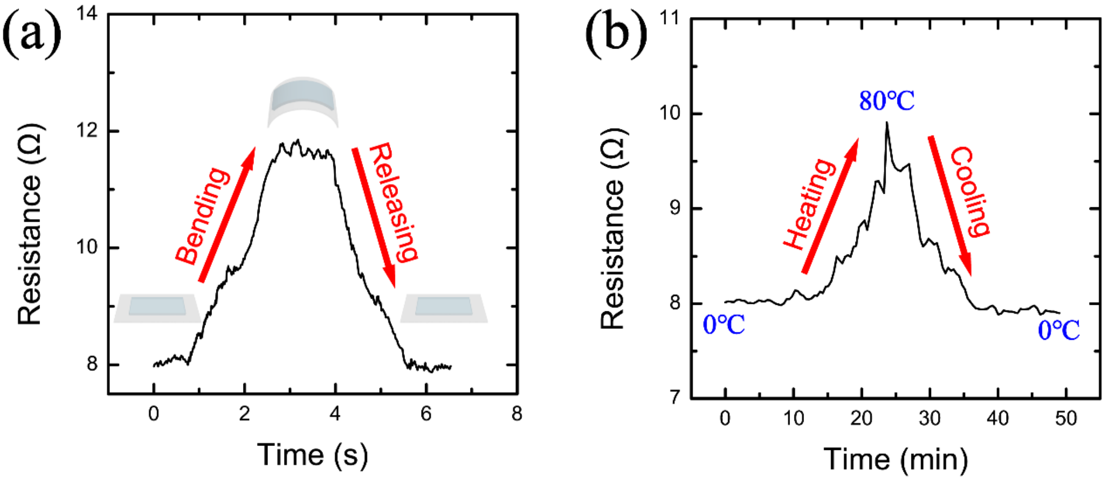

Figure S4. The stability of the electrode under different external stimuli, (**a**) bending test, (**b**) temperature variation test.

6. Statistics on the number and density of Ag nanosheets

The electrodes with different mass ratios of PDMS and Ag are divided into 64 same areas (15 × 15 μm²/unit), and the number of Ag nanosheets in the areas is obtained using the counting method, as shown in Figure S5. In addition, the density of Ag nanosheets is calculated, as shown in Table S2.

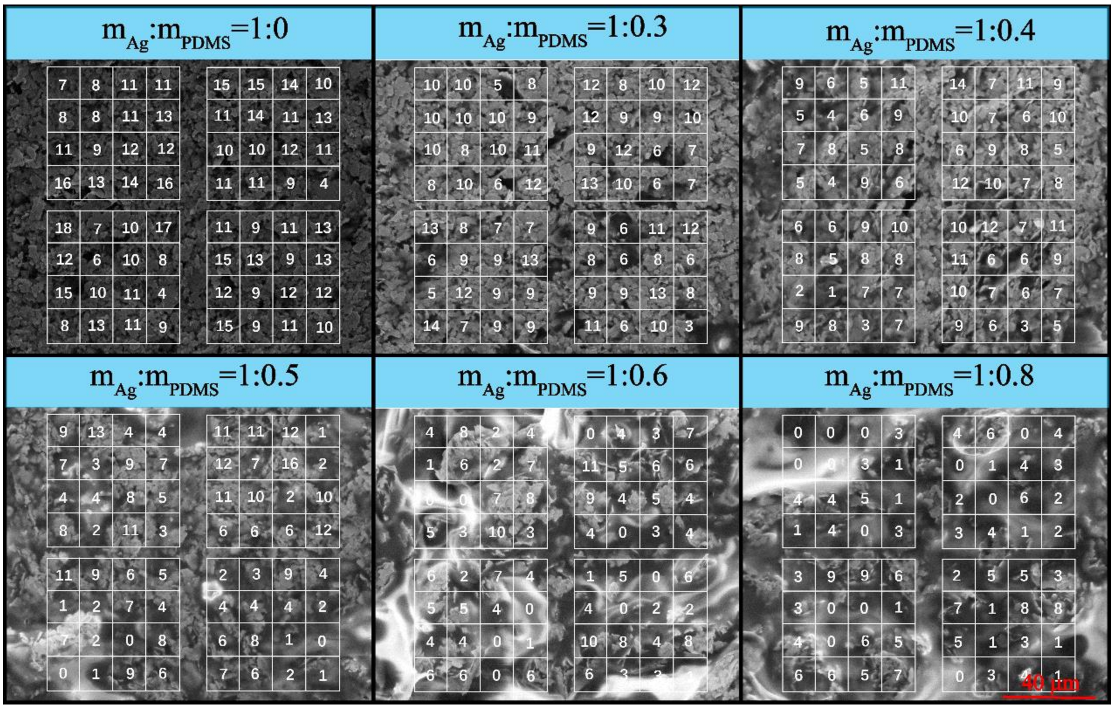

Figure S5. The statistics on the number of Ag nanosheets by counting method.

Table S2. The statistics on the density of Ag nanosheets.

$m_{silver\ glue}$:m_{PDMS}	Number (/14,400 μm²)	Density (N/μm²)
1:0	714	0.04958
1:0.3	540	0.04028
1:0.4	475	0.03299
1:0.5	367	0.02549
1:0.6	268	0.01861

1:0.8	198	0.01375

7. Derivation of the contact point theory

The relationship between the conductivity of electrode and Ag nanosheets can be expressed by the contact point theory as follows:

$$\sigma = \frac{1}{R_0}\beta N \quad (1)$$

Where σ and N are the conductivity of the electrode and the number of the contact points between the adjacent Ag nanosheets, respectively, and β is a proportional constant.

Since the number of contact points decreases as the strain increases, and the rate of decrease is related to the number of contact points, the following relationship can be expressed as:

$$\frac{dN}{d\varepsilon} = -\alpha N \quad (2)$$

Where ε is the strain value and α is a proportional constant. Then, Equation (1) and Equation (2) are solved jointly, and the following relationship can be obtained:

$$\ln\frac{R}{R_0} = \alpha\varepsilon + b \quad (3)$$

Since there is a negative correlation between strain value and the density of Ag nanosheets during the stretching process, Equation (3) can be converted into a relationship between the resistance and the density of Ag nanosheets as follows:

$$\ln\frac{R}{R_0} = \alpha'\frac{D}{D_0} + b' \quad (4)$$

Where D and D_0 are the density and initial density of Ag nanosheets, respectively. Thus, the contact point theory suggests a linear relationship between the relative density (D/D_0) of the Ag nanosheets and the logarithm of the relative resistance (R/R_0) of the electrode.

8. Derivation of the tunneling theory

According to the tunneling theoretical model, the resistance of the electrode during stretching can be expressed as:

$$R_t = (\frac{L}{T})(\frac{8hs}{3a^2\gamma e^2})\exp(\gamma s) \quad (5)$$

Where

$$\gamma = \frac{4\pi}{h}\sqrt{2m\varphi} \quad (6)$$

In this equation, L is the number of Ag nanosheets in a single conductive pathway, T is the number of conductive pathways, h is Planck's constant, s is the minimum distance between Ag nanosheets, α^2 is the effective cross-sectional area where the tunneling effect occurs, e is the electron charge, m is the electron mass, φ is the height of the barrier between adjacent Ag nanosheets.

When strain occurs, the relative change of the tunneling resistance R_t/R_0 is:

$$\frac{R_t}{R_0} = \frac{T_0 s}{T s_0} \exp[\gamma (s - s_0)] \tag{7}$$

Where T_0 and s_0 are the number of the conductive pathways and the minimum distance between Ag nanosheets before strain occurs (ε=0), and s can be represented by $s_0(1+\varepsilon)$.

During stretching process, the number of conductive pathways decreases with the increasing strain, and the change rate is related to the number of conductive pathways, which can be expressed as:

$$\frac{dT}{d\varepsilon} = -(A + B\varepsilon + C\varepsilon^2 + \cdots)T \tag{8}$$

Where A, B and C are constant coefficient. In this study, in order to simplify the calculation model, only the first two items are considered as follows:

$$\frac{dT}{d\varepsilon} = -(A + B\varepsilon)T \tag{9}$$

By combining Equations (7)–(9), the relationship between the tunneling resistance and strain can be obtained as follows:

$$\ln(\frac{R_t}{R_0}) = A'\varepsilon^2 + B'\varepsilon + \ln(1+\varepsilon) \tag{10}$$

Since the values of ln(1+ε) and ε are close when ε < 50%, ε can be used to replace ln(1+ε), thus simplifying to the following form:

$$\ln(\frac{R_t}{R_0}) = a\varepsilon^2 + b\varepsilon \tag{11}$$

Different from the contact point theory, which reflects the state quantity of resistance, the tunneling effect theory reflects the resistance change during the stretching process. In Equation (11), $a\varepsilon^2$ represents the tunneling resistance term during stretching process, and $b\varepsilon$ represents the contact resistance term of the electrode. Therefore, the total resistance of the electrode can reflect both the contact state inside the electrode and the change process of resistance.

9. Statistics on the size of Ag nanosheets

In the SEM image of PDMS-Ag electrode, an area of 40 × 40 μm^2 in size was selected as the statistical area, and the radius (the longest radius in the same Ag nanosheet was taken as the statistical radius) of each Ag nanosheets in this area was obtained by direct measurement, as shown in Figure S6a. The measured Ag nanosheet radii were sorted, and the statistical results are shown in Figure S6b. According to the statistical results, the average radius of the Ag nanosheet is calculated to 3.912 μm.

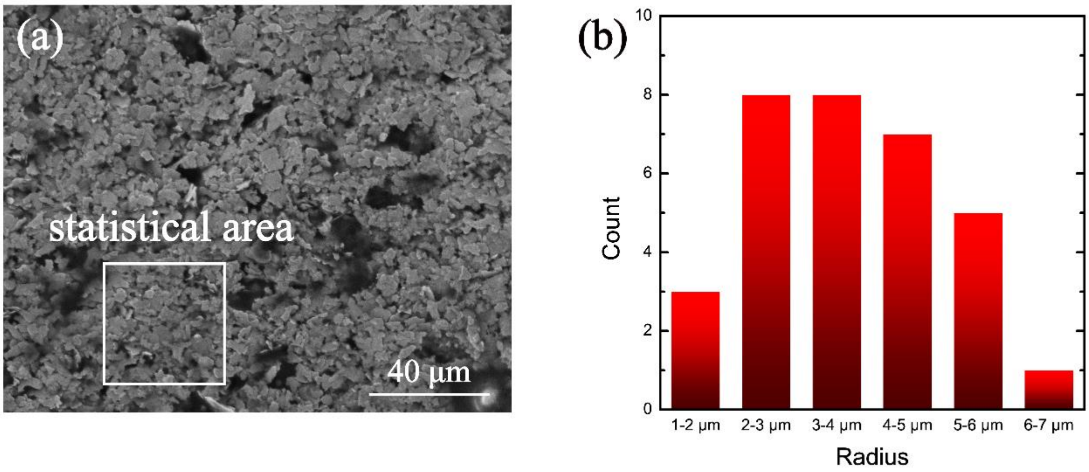

Figure S6. The statistical size of the Ag nanosheet. (**a**) SEM image of the statistical area. (**b**) The statistical results of the Ag nanosheet radii.

10. Modeling process of electrodes through the finite element simulation

The physical model of the PDMS-Ag composite electrode is established by combining the statistics of the density and size of the Ag nanosheets (Figure S7a), and an electric potential of 0.1 V is applied to the model, the potential distribution is shown in Figure S7b. It can be observed that the potential decreases slowly in which the Ag nanosheets are dense and decreases quickly in which the Ag nanosheets are loose.

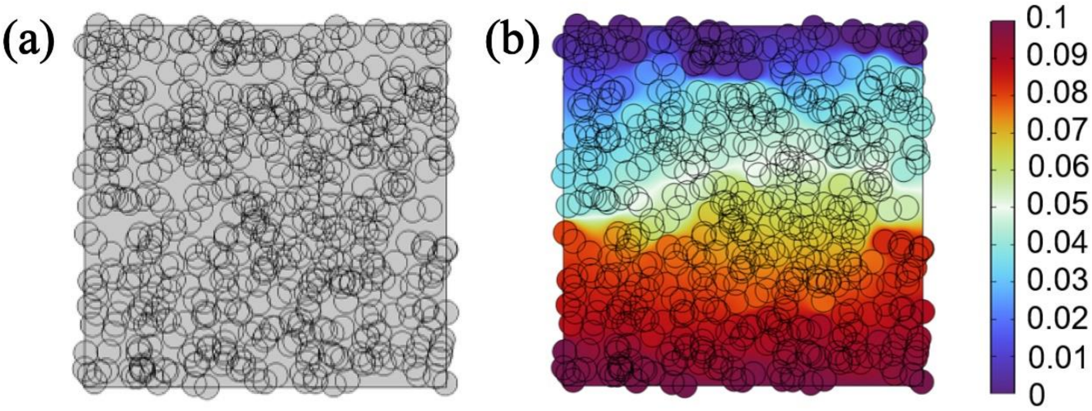

Figure S7. Modeling process of the electrode through the finite element simulation. The schematic of (**a**) model building and (**b**) potential distribution.
